# Detailed Enzyme Kinetics in Terms of Biochemical Species: Study of Citrate Synthase

**DOI:** 10.1371/journal.pone.0001825

**Published:** 2008-03-19

**Authors:** Daniel A. Beard, Kalyan C. Vinnakota, Fan Wu

**Affiliations:** Biotechnology and Bioengineering Center and Department of Physiology, Medical College of Wisconsin, Milwaukee, Wisconsin, United States of America; University of Arizona, United States of America

## Abstract

The compulsory-ordered ternary catalytic mechanism for two-substrate two-product enzymes is analyzed to account for binding of inhibitors to each of the four enzyme states and to maintain the relationship between the kinetic constants and the reaction equilibrium constant. The developed quasi-steady flux expression is applied to the analysis of data from citrate synthase to determine and parameterize a kinetic scheme in terms of biochemical species, in which the effects of pH, ionic strength, and cation binding to biochemical species are explicitly accounted for in the analysis of the data. This analysis provides a mechanistic model that is consistent with the data that have been used support competing hypotheses regarding the catalytic mechanism of this enzyme.

## Introduction

While the study of the catalytic kinetics of enzymes represents one of the most established and well documented fields in biochemical research, the impact of biochemical state (pH, ionic strength, temperature, and certain cation concentrations) is typically not formally accounted for in kinetic studies [1], [2]. *In vitro* experiments using purified proteins and controlled substrate concentrations to characterize enzyme kinetics are conducted under conditions that do not necessarily match the physiological environment, but are determined based on a number of factors, including the requirements of the assays used to measure the kinetics. Therefore it is difficult to compare results obtained from different studies and to use available kinetic data to predict *in vivo* function without ambiguity.

The need for credible validated models (such as enzymatic rate laws and associated parameter values) for the individual components of a given biochemical system is apparent in developing simulations of cellular biochemical systems. For example, simulations of metabolic systems, such as the glycolytic pathway in yeast [3], skeletal muscle [4], [5], and mammalian red blood cells [6], [7], are based on integrating the individual components together. Simulations of other cellular systems, such as signaling networks and membrane electrophysiology, are also based on kinetic models for mechanisms of relevant individual enzymes and transporter proteins. To apply these models to simulate and predict cellular behavior, they must not only match the available data but also properly account for biochemical state.

Outlining these and other issues in somewhat greater detail, the following specific challenges associated with interpreting *in vitro* kinetic data must be overcome to make optimal use of them.

1. While a great deal of high quality data may be available for a particular enzyme, much of these data were obtained in the 1960's and 1970's when tools for proper analysis of the data were not available. As a result, the reported kinetic parameter values (typically obtained from double reciprocal plots of inverse flux versus inverse substrate [8]) may not optimally match the reported data.
2. Data on biochemical kinetics are typically obtained under nonphysiological pH and ionic conditions. Therefore the reported kinetic constants must be corrected to apply to simulations of physiological systems.
3. A third problem related to the second is that kinetic constants are associated with apparent mechanisms that operate on biochemical reactants, which are sums of biochemical species [2]. The result is that the reported mechanisms and associated parameter values are dependent on biochemical state and not easily translated to apply to different biochemical states or to simulations in which the biochemical state changes.
4. The reported kinetic mechanisms and parameters are often not constrained to match the thermodynamic data for a given reaction. Since the basic thermodynamics of a given reaction is typically characterized with greater precision than the kinetics of an enzyme catalyzing the reaction, putative kinetic mechanisms should be constrained to match the biochemical reaction thermodynamics.

We propose addressing and correcting these problems by posing reaction mechanisms in terms of species and ensuring that mechanisms properly account for thermodynamics. This basic approach was first introduced by Frieden and Alberty [9], yet has received little attention. Here, we reanalyze legacy data from a variety of sources of kinetic data on citrate synthase. Rather than estimating apparent Michaelis-Menten parameter values from slopes of double reciprocal plots, we use nonlinear curve fitting to simultaneously estimate parameter values from several sets of data from kinetic studies on specific isoforms of the enzyme. Through this analysis we are able to show that data used to support competing models of the mechanism for this enzyme are all consistent with the compulsory-order ternary-complex mechanism. In addition, certain conclusions drawn from the original studies are shown to be not consistent with the data presented in these studies.

To perform this analysis on citrate synthase it is first necessary to derive the general rate law (quasi-steady flux expression) for the compulsory-order ternary-complex mechanism that can account for potential nonproductive binding of inhibitors at any of the four distinct enzyme states. Although the derivation of quasi-steady rate laws for multi-state catalytic mechanisms is a rich and established field, the general form for this mechanism with potential inhibition at any site, has not previously been presented. Therefore it is expected that this expression will be useful in the analysis of a number of other two-substrate two-product catalytic mechanisms.

## Methods and Results

### Kinetic equations for compulsory-order ternary-complex enzyme mechanism

**Figure 1:**
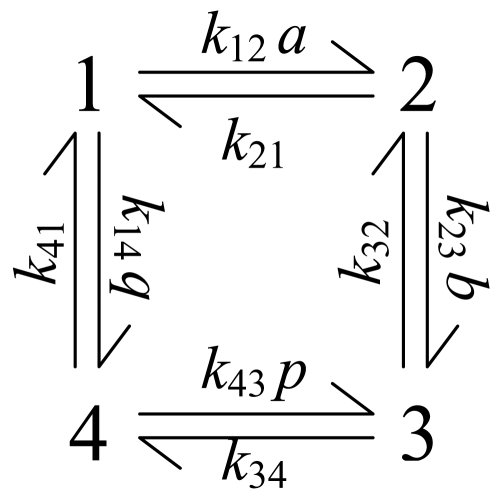
Basic compulsory-order ternary-complex mechanism. The basic ordered mechanism for the general reaction 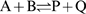, with *a* = [A], *b* = [B], *p* = [P], and *q* = [Q] is illustrated. The four states refer to unbound enzyme (state 1), enzyme-substrate A complex (E·A, state 2), enzyme-substrate A-substrate B complex (E·AB, state 3), and enzyme-product Q complex (E·Q, state 4). The four steps of the catalytic cycle are detailed in Equation (1).

The basic compulsory-order ternary-complex mechanism, also called the ordered bi-bi mechanism, is illustrated in Figure 1 for the general reaction 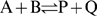. The mechanism involves four enzyme state transitions:
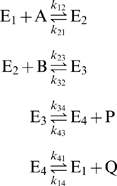
(1)where each state transition is assumed to proceed by mass action [10], [11]. Here E_1_ represents free (unbound) enzyme; E_2_ represents the complex formed between enzyme and the species A, which binds first; E_3_ is the ternary complex that represents enzyme bound to both substrates or both products; and E_4_ represents the complex formed between enzyme and the species Q. In Figure 1 the substrate and product concentrations are denoted *a* = [A], *b* = [B], *p* = [P], and *q* = [Q] and the reactant concentrations are incorporated into apparent mass-action rate constants for the state transitions between enzyme states 1, 2, 3, and 4.

From the four-state diagram of Figure 1, the expression for the steady-state flux through the reaction can be obtained from diagrammatic method of King and Altman [12]. The flux *J* may be expressed
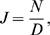
(2)where
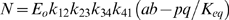
(3)and
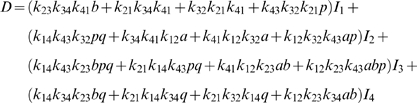
(4)The constant *K_eq_* is the equilibrium constant for the reaction; *E_o_* = *E_1_*+*E_2_*+*E_3_*+*E_4_* is the total enzyme concentration; and the *I_i_* factors in Equation (4) account for nonproductive binding (inhibition) of inhibitors to each of the enzyme states. These inhibition factors are computed
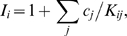
(5)where *K_ij_* is the binding constant for nonproductive binding of species *j* to enzyme state *i* and *c_j_* is the concentration of species *j*.

Defining
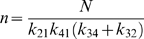
(6)and
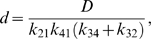
(7)the flux is *J* = *n*/*d*, where the numerator and denominator expressed in terms of kinetic constants are
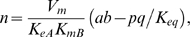
(8)and
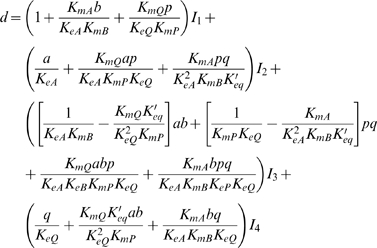
(9)Here the kinetic constants are defined
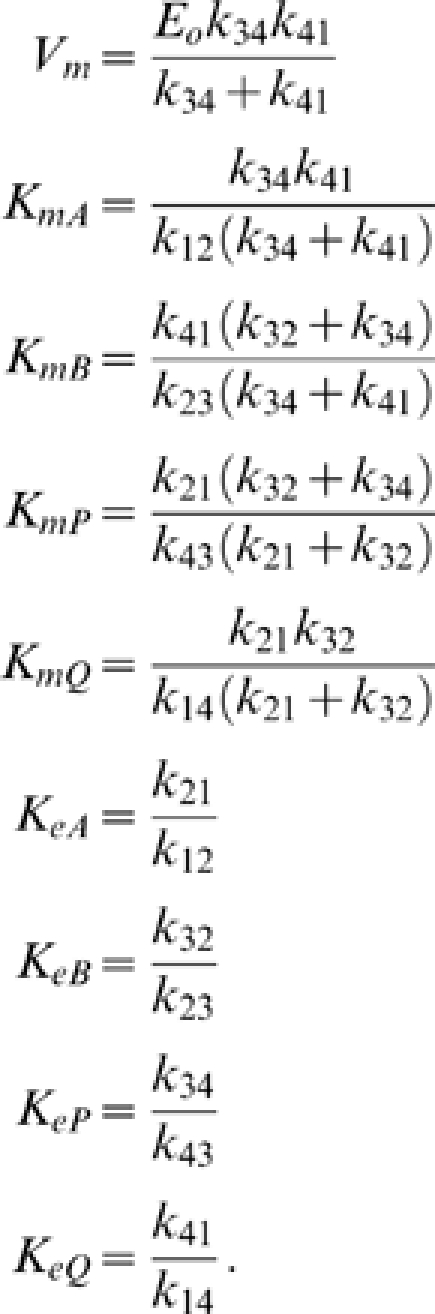
(10)


Expressing the steady-state kinetics in terms of these parameters, only the *V_m_* parameter, which has units of mass per unit time per unit volume, has units that include time. All other parameters have units of concentration (mass per unit volume). In addition, the eight concentration parameters cannot vary independently. For example we can compute *K_eQ_* in terms of the other parameters if the equilibrium constant of the reaction is known:
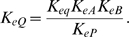
(11)


The novelty of the expression of Equation (9) for the denominator of the flux expression is that inhibitive binding at each enzyme state is considered. It is expected that a wide class catalytic mechanisms for two-substrate two-product reactions will conform to this general form.

Here we apply this general form to the analysis of data from citrate synthase to determine kinetic parameters for several isoforms of this enzyme and to elucidate the mechanisms behind inhibition by products and other species not part of the overall chemical reaction.

### Model of citrate synthase

Citrate synthase is the first step in the oxidation of acetyl-CoA in the citric acid cycle. The reference chemical reaction, is
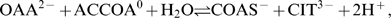
(12)where the abbreviations for the biochemical species are listed in Table 1. The biochemical reaction, involving biochemical reactants that are sums of species is
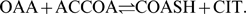
(13)


Note that we have introduced the convention that the charge of a species appears as a superscript, even when the charge is zero. This convention conveniently differentiates between, for example, the species ACCOA^0^ and the reactant ACCOA.

**Table 1:** Thermodynamic Parameter Values for Citrate Synthase (298.15 K, 1 M reactants, I = 0.17 M, P = 1 atm).

Reactant	Abbreviation	Reference species	*Δ*_f_G_o_ (kJ/mol)	Ion-bound species	pK
water	H_2_O	H_2_O	−235.74	-	-
coenzyme A	COASH	COAS^−^	−0.72	COASH^0^	8.13
acetyl-co-enzyme A	ACCOA	ACCOA^0^	−178.19	-	-
oxaloacete	OAA	OAA^2−^	−794.41	MgOAA^0^	0.0051^a^
citrate	CIT	CIT^3−^	−1165.59	HCIT^2−^	5.63
				MgCIT^−^	3.37^a^
				KCIT^2−^	0.339^a^
adenosine triphosphate	ATP	ATP^4−^	−2771.00	HATP^3−^	6.59
				MgATP^2^	3.82^a^
				KATP^3−^	1.87^a^
adenosine diiphosphate	ADP	ADP^3−^	−1903.96	HADP^2−^	6.42
				MgADP^−^	2.79^a^
				KADP^2−^	1.53^a^
adenosine monophosphate	AMP	AMP^2−^	−1034.66	HAMP^−^	6.22
				MgAMP^0^	1.86^a^
				KAMP^−^	1.05^a^
succinyl-coenzyme A	SCOA	SCOA^−^	−507.55	HSCOA^0^	3.96

All values from [13] unless otherwise noted.

a NIST database 46: Critical Stability Constants.

The standard Gibbs free energy is computed [13]:
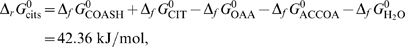
(14)where the basic thermodynamic data are listed in Table 1. The equilibrium constant for reaction in Equation (12) is computed from the standard Gibbs free energy
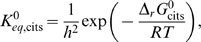
(15)where we have introduced the definition *h* = 10^−pH^ and this equilibrium constant explicitly accounts for pH. Therefore *K* ^0^ *_eq_*_,cits_ represents the equilibrium ratio of [COAS^−^][CIT^3−^]/[OAA^2−^][ACCOA^0^].The relationships between species and reactant concentrations depend on the pH and concentration of metal ions that reversibly bind to biochemical species. To compute species concentrations and the apparent equilibrium constant and Gibbs free energy, we introduce binding polynomials for reactants and other species that we consider in our model of citrate synthase kinetics:
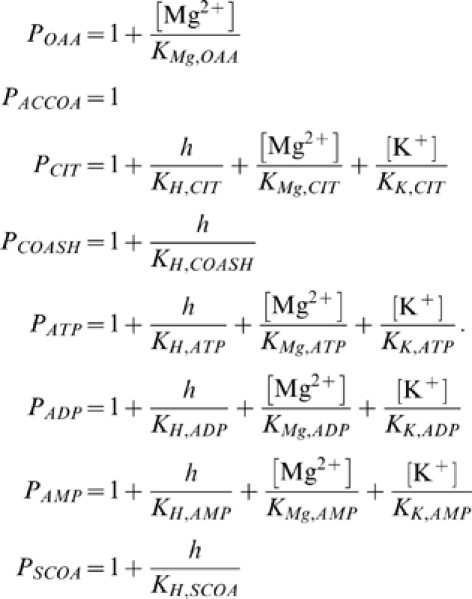
(16)


These polynomials include terms for H^+^-, Mg^2+^-, and K^+^-bound states [13]. Note that only states that are expected to be significant in the pH and ionic range studied are included in these calculations. Therefore some binding polynomials do not include terms for all possible cation-bound states. Given these forms of the binding polynomials, the relationships between the reference species concentrations and the reactant concentrations are
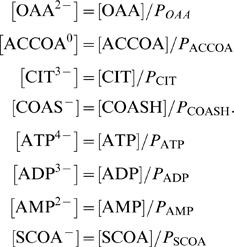
(17)


Under typical physiological conditions (pH = 7, [K^+^] = 120 mM, and [Mg^2+^] = 1 mM) the fraction of these reactants present as their unbound reference species may be smaller than 10%. For example, the molar fraction of ATP^4−^, HATP^3−^, MgATP^2−^, KATP^3−^, predicted based on the parameters in Table 1 are approximately 0.085, 0.024, 0.32, and 0.57.

The apparent equilibrium constant for the biochemical reaction is computed as a function of pH, [K^+^], and [Mg^2+^]
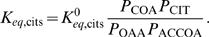
(18)


Citrate synthase is believe to operate by the compulsory-order ternary-complex mechanism, although investigations have lead to proposing more complex behavior, involving cooperativity and random order and dead-end binding of substrates [14]. Here we postulate the standard compulsory-order ternary-complex mechanism derived above can explain the kinetic behavior of citrate synthase with substrate and products identified as: *a* = [OAA^2−^], *b* = [ACCOA^0^], *p* = [COAS^−^], *q* = [CIT^3−^]. The specific mechanism proposed is:
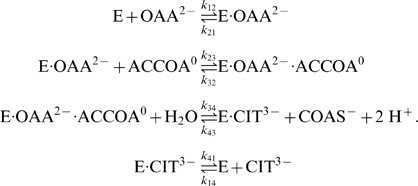
(19)


As an alternative mechanism, we could postulate that the second and third reactions have the form
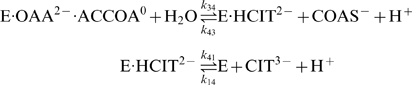
(20)where both the second and third reactions generate protons. Based on the data analyzed we are not able to distinguish between these models. The analysis here applies to the mechanism of Equation (19). In this case only the third reaction (in which hydrogen ion explicitly appears) depends on pH. Since *K_eP_*, the equilibrium constant for the third reaction, depends on pH while the others do not, we compute *K_eP_*, as a function of the equilibrium constant for the reference reaction
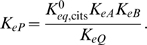
(21)The rate constant *k*_43_ is assumed to depend on pH according to the formula *k*_43_ = (*h*/10^−7^)^2^ *k*′_43_ where *k*′_43_ is independent of pH. Therefore the kinetic constant *K_mP_* is defined to depend on pH as
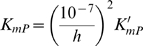
(22)where *K*′*_mP_* is a kinetic constant that is independent of pH. In addition to the pH-dependency of the kinetic constants, the overall enzyme activity is assumed to depend on pH, with the numerator of the flux expression taking the form:
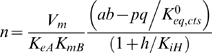
(23)which is used to reproduce the pH dependency observed by Shepherd and Garland [15]. Equation (23) assumes that the enzyme is a monobasic acid, with dissociation constant *K_iH_*.

Previous studies have revealed that a number of substances, including succinyl-coenzyme A and adenine nucleotides, act as inhibitors of citrate synthase. Our analysis of kinetic data on citrate synthase from rat liver and bovine heart (see below) revealed that ATP, ADP, and AMP inhibit the enzyme by forming unproductive complexes with enzyme state 2. Because we were able to obtain less data on SCOA inhibition than on adenine nucleotide inhibition, we were not able to elucidate the site of SCOA binding: models assuming binding at either state 1 or state 2 are equally well able to explain the observed data. Since the adenine nucleotide inhibition was determined to occur at enzyme state 2, here we parameterize the model assuming that SCOA binds to this complex as well. Based on this formulation of the model, the inhibition term *I_2_* is
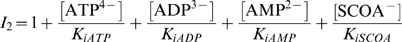
(24)and inhibition at other complexes is not considered: *I_1_* = *I_3_* = *I_4_* = 1.

### Analysis of data for the kidney enzyme from rat

**Figure 2:**
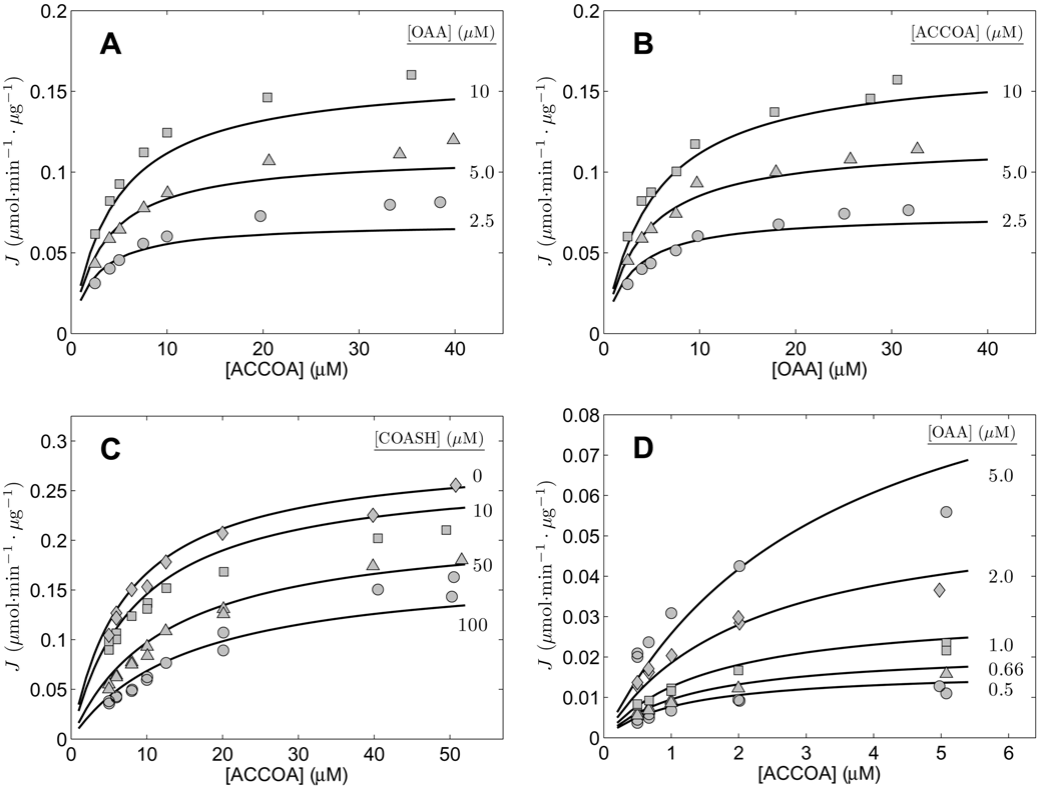
Fits to kinetic data from [14] on the forward operation of kidney enzyme. Measured flux as a function of substrate concentrations was obtained from Figures 2, 3, 6, 7, and 9 of [14]. Initial fluxes (µmol of COASH (or CIT) synthesized per minute per µg of enzyme) measured at the substrate concentrations indicated in the figures. For A, B, and D, the initial product (CIT and COASH) concentrations are zero. C. Flux measured with COASH added in various concentrations to investigate the kinetics of product inhibition. All data were obtained at pH = 8.1 at 28°C. Model fits are plotted as solid lines.

**Figure 3:**
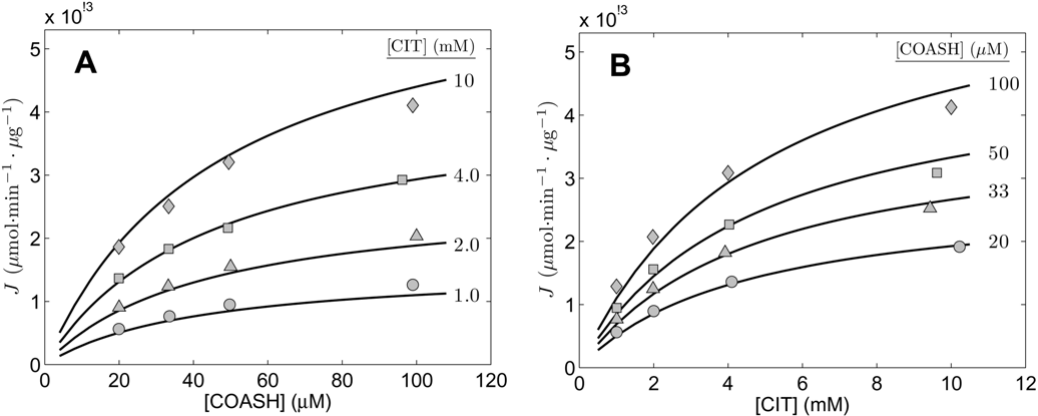
Fits to kinetic data from [14] on the reverse operation of kidney enzyme. Measured reverse flux as a function of concentrations of CIT and COASH was obtained from Figures 4 and 5 of [14]. Initial fluxes (µmol of COASH (or CIT) synthesized per minute per µg of enzyme) measured at the substrate concentrations indicated in the figures. All data were obtained at pH = 8.1 at 28°C. Model fits are plotted as solid lines.

Matsuoka and Srere reported a comprehensive study on the forward and reverse kinetics of citrate synthase from rat kidney [14] that is useful in identifying the kinetic parameters for this enzyme. Data used here are plotted in Figures 2 and 3.

Figure 2 plots data on the forward reaction flux as functions of the concentrations of substrates OAA and ACCOA, corresponding to data from Figures 2, 3, 6, 7, and 9 of Matsuoka and Srere [14]. Figure 2A plots flux in units of µmol of COASH (or CIT) synthesized per minute per µg of enzyme as a function of [ACCOA] at different concentrations of [OAA] while Figure 2B plots flux versus [OAA] at different concentrations of [ACCOA], as indicated in the figure. Product inhibition by COASH is illustrated in Figure 2C, which plots flux versus [ACCOA] at [OAA] = 0.5 mM and concentrations of [COASH] ranging from 0 to 100 µM. The data plotted in Figure 2D are analogous to that of Figure 2A, with the difference that the range of substrate concentrations in Figure 2D are significantly lower than in Figure 2A.

Figures 6 and 7 Matsuoka and Srere [14] report data obtained for low concentrations of ACCOA, while Figures 2 and 3 report data at the higher concentration range. However, where the concentration ranges intersect, the reported fluxes (in units of nmole/min) are approximately five times higher in their Figures 2 and 3 than in their Figures 6 and 7. Since the assays in their Figures 2 and 3 were carried out in a 1 ml cuvette and the assays in their Figures 6 and 7 were carried out in a 5 ml cuvette, we have deduced that the data in Figures 6 and 7 were normalized to the cuvette size. This finding is verified based on their reported estimated *V_max_* values, which are consistent with this scaling. Therefore, we have scaled the data of Figures 6 and 7 of Matsuoka and Srere [14] (replotted here in panel 2D) by a factor of five compared to the other figures.

**Figure 4:**
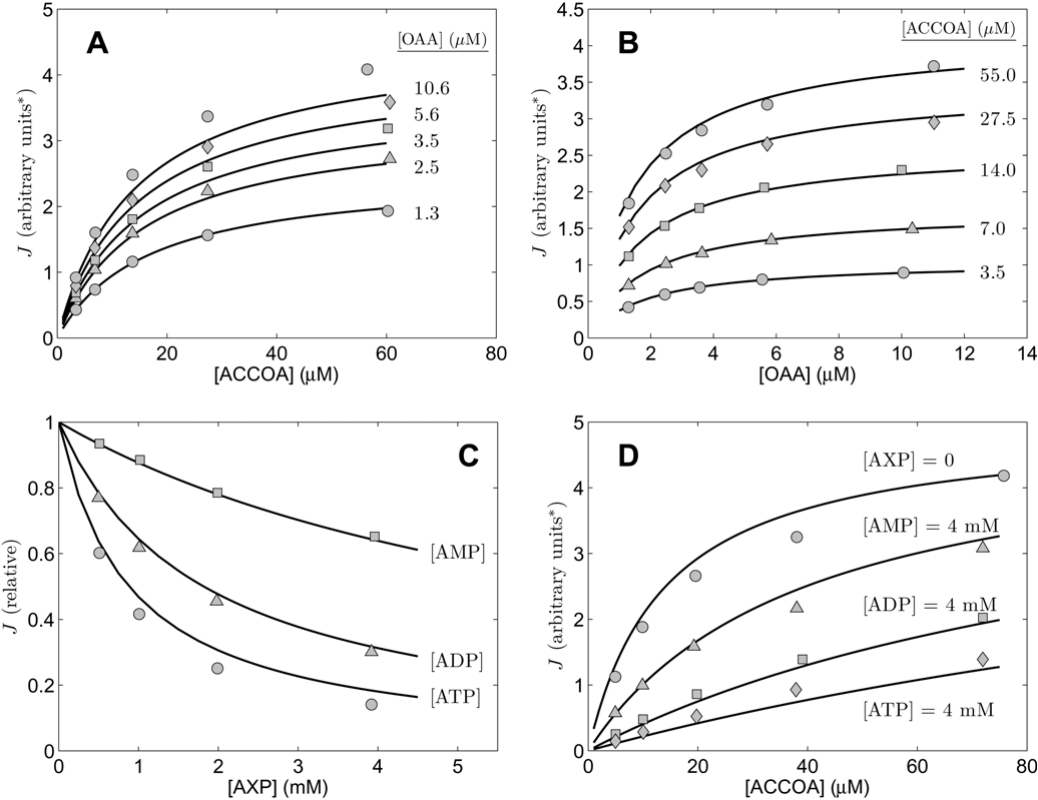
Fits to kinetic data from [15] on the forward operation of liver enzyme. Measured flux in arbitrary units was obtained from Figures 1,2,5, and 6 of [15]. For all cases the product (CIT and COASH) concentrations are zero and total substrate and inhibitor concentrations are indicated in the figure. A and B report data obtained with no inhibitors present. C. The relative activity (normalized to its maximum) of the enzyme is plotted as functions of [ATP], [ADP], and [AMP] measured at [ACCOA] = 11 µM and [OAA] = 1.9 µM. D. The measured flux is plotted as a function of [ACCOA] at [OAA] = 34 µM with ATP, ADP, and AMP present as indicated in the figure. All data were obtained at pH = 7.4 at 25°C. Model fits are plotted as solid lines.

**Figure 5:**
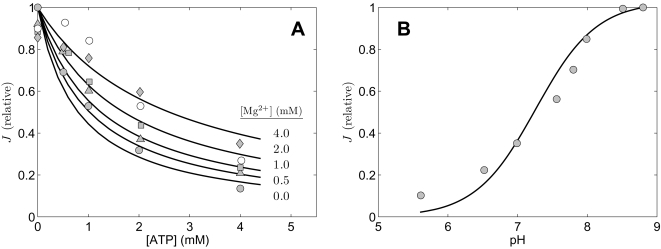
Impact of [Mg^2+^] and pH on liver enzyme. Measured flux in arbitrary units was obtained from Figures 13 and 14 of [15]. A. The relative activity (normalized to its maximum) of the enzyme is plotted as functions of [ATP] at [Mg^2+^] = 0 mM (shaded circles), 0.5 mM (shaded triangles), 1.0 mM (shaded squares), 2.0 mM (open circles), and 4.0 mM (diamonds). B. Relative activity is plotted as a function of pH. Substrate concentations are [ACCOA] = 21 µM and [OAA] = 8.6 µM. All data were obtained at 25°C. pH is fixed a 7.4 for A. Model fits are plotted as solid lines.

Data on the reverse flux of the enzyme are plotted in Figure 3. Figures 3A and 3B correspond to Figures 4 and 5, respectively, of Matsuoka and Srere [14], where reverse flux is reported over a range of [COASH] and [CIT] values.

All experiments for the data of Figures 2 and 3 were conducted at pH = 8.1 at 28°C. We assume and overall ionic strength of 0.17 M, and [K^+^] = 100 mM, and [Mg^2+^] = 0. The complete set of data from Figures 2 and 3 were used to estimate parameter values for the kidney enzyme. Specifically, we use these data to estimate *V_m_*, *K_mA_*, *K_mB_*, *K*′*_mP_*, *K_mQ_*, *K_eA_*, *K_eB_*, and *K_eQ_*. The parameter values associated with the best fits to the data are listed in Table 2 and the corresponding model predictions are plotted as solid lines in the figures. The agreement between the data and the model is satisfactory, with values of the eight estimated parameters sensitive to the observed data. Estimated sensitivity coefficients (defined below) are given in parenthesis for each parameter value in Table 2.

**Table 2:** Kinetic Parameter Values for Citrate Synthase.

Parameter	Rat kidney (based on data from [14])	Rat liver (based on data from [15])	Bovine heart (based on data from [17])
*V_max_* (µmol·min^−1^·µg^−1^)	0.335 (1.26)	—	—
*K_mA_* (µM)	8.17 (0.39)	1.37 (0.12)	2.01 (0.27)
*K_mB_* (µM)	7.36 (0.47)	14.4 (0.31)	8.66 (0.10)
*K*′*_mP_*(µM)	0.15 (0.004)	—	—
*K_mQ_* (mM)	4.63 (0.033)	—	—
*K_eA_* (µM)	0.91 (0.31)	1.62 (0.08)	0.90 (0.003)
*K_eB_* (µM)	29.7 (0.006)	—	—
*K_eQ_* (mM)	3.93 (0.02)	—	—
*K_iATP_* (µM)	—	37.3 (0.28)	73.0 (0.24)
*K_iADP_* (µM)	—	135.4 (0.28)	—
*K_iAMP_* (µM)	—	992.3 (0.27)	—
*K_iSCOA_* (µM)	—	—	74.1 (0.10)
*K_iH_* (µM)	—	0.055 (0.53)	—

Based on their data, Matsuoka and Srere report estimates for the kinetic parameters, some of which can be compared to those estimated here. It is not surprising that the estimates of *V_m_* (estimated at high ACCOA concentrations by Matsuoka and Srere to be 0.316 µmol·min^−1^·µg^−1^ and estimated here to be 0.336 µmol·min^−1^·µg^−1^) are similar. In addition, Matsuoka and Srere's reported estimates kinetic parameter values for the “medium” ACCOA concentration range (5–50 µM) are *K_mA_* = 5 µM, *K_mB_* = 4.5 µM, *K_mP_* = 39 µM, *K_mQ_* = 3 mM, *K_eA_* = 5 µM, and *K_eQ_* = 4.3 mM. These values can be directly compared to our estimates of *K_mA_* = 8.227 µM, *K_mB_* = 7.402 µM, *K_mP_* = 24.72 µM (at pH 8.1), *K_mQ_* = 4.548 mM, *K_eA_* = 0.8879 µM, and *K_eQ_* = 3.618 mM. (The parameters *K_eB_* and *K_eP_* are not directly comparable to those estimated by Matsuoka and Srere because they are defined differently in the two studies.)

Parameter estimates from our study correspond to species, while those from Matsuoka and Srere correspond to reactants; thus the applied models are not exactly identical. More significantly, we performed our model fits by simultaneously matching all of the data in Figures 2 and 3 to the model using a single set of parameter estimates rather than estimating kinetic constants from the slopes of double reciprocal plots. The result is that we are able to explain the data based on a single mechanism operating at all observed concentration ranges, while Matsuoka and Srere reported different estimates of apparent *V_m_* and other kinetic constants operating at low (<5 µM), medium (5–50 µM) and high (>50 µM) concentrations of ACCOA.

Matsuoka and Srere speculated that their findings may be explained by the existence of cooperativity in ACCOA binding or by a random binding mechanism. Yet while we are able to explain the high-ACCOA data (Figure 2 of [14]) and the low-ACCOA data (Figure 7 of [14]), our model is not able to reproduce the data presented in Figure 8 of [14], which shows a discontinuity in the slope of the double reciprocal plot of *V* ^−1^ versus [ACCOA]^−1^. In fact, we are at a loss to explain Figure 8 of Matsuoka and Srere because the concentration ranges explored in their Figures 2 and 7 overlap and extend beyond the range explored in their Figure 8. The data of their Figure 2 and 7, when plotted together, all fall on the same straight line. Therefore it is possible that an incorrect scaling was applied to a portion of the data presented in [14]. In any case, it is apparent that there exists an inconsistency in the reported data. Since to our knowledge no other study reproduced the slope discontinuity reported in [14], we chose to leave this data set out of our analysis.

The largest disagreement between our estimated kinetic constants and those estimated by Matsuoka and Srere for the medium ACCOA concentration range is in the estimate of *K_eA_*, the dissociation constant for OAA. Yet our estimate of 0.8879 µM is in agreement with an independent study by Srere [16] that estimated *K_eA_* to be 0.6 µM for citrate synthase obtained from pig heart, providing further validation of our proposed model.

### Analysis of data for the liver enzyme from rat

A detailed kinetic study on citrate synthase obtained from rat liver [15] provides an opportunity to check the basic model developed above based on and independent data set and to assess the impact of inhibition of the enzyme by ATP, ADP, and AMP. While we do not expect the kinetic constants to be the same for the liver isoform as for the kidney enzyme studied above, we hypothesize that the basic kinetic mechanism is the same for both isoforms.

**Figure 6:**
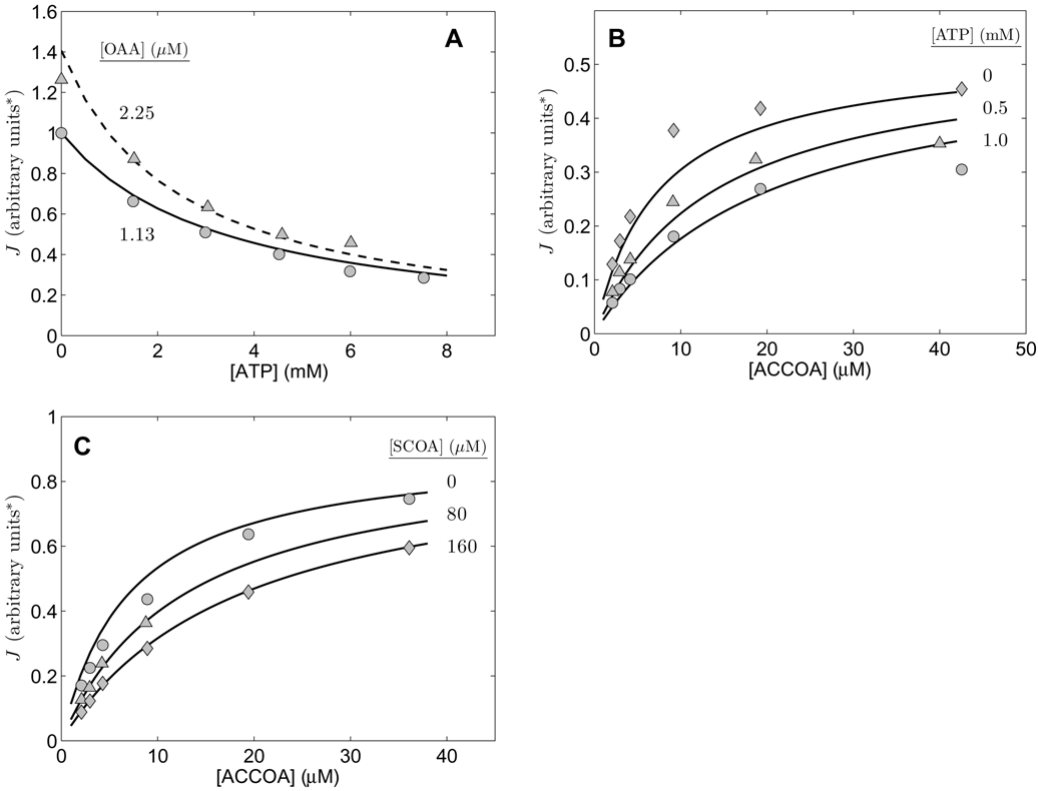
Inhibition of cardiac enzyme. Measured flux in arbitrary units was obtained from Figures 1 and 2 of [17]. A. Flux is plotted as a function inhibitor ATP concentration for [ACCOA] = 16 µM and [OAA] = 1.13 and 2.25 µM. B. Flux is plotted as a function of [ACCOA] at [OAA] = 5 µM at three different concentrations of ATP indicated in figure. C. Flux is plotted as a function of [ACCOA] at [OAA] = 3.1 µM at three different concentrations of SCOA indicated in figure. All data were obtained at pH = 7.4 at 21°C. Model fits are plotted as solid lines.

Figures 4 and 5 plot data obtained from Figures 1, 2, 5, 6, 13, and 14 of Shepherd and Garland [15], all corresponding to the forward reaction flux with no product present in the assays. Experiments were conducted at pH = 7.4 (except those plotted in Figure 5B) at 25°C. Again, we assume and overall ionic strength of 0.17 M, and [K^+^] = 100 mM, and [Mg^2+^] = 0. The experiments of Figures 4A and 4B are analogous to those of Figures 2A and 2B, in which the substrate concentrations are varied to obtain estimates of *K_mA_*, *K_mB_*, and *K_eA_*. (Since the total mass of enzyme used in these experiments is not specified, it is not possible to estimate *V_m_* from these data.)

Experimental data plotted in Figure 4C and 4D provide information on inhibition of the enzyme due to binding of ATP, ADP, and AMP. Figure 4C plots the inhibition (as relative activity normalized to its maximum) as functions of [ATP], [ADP], and [AMP] measured at [ACCOA] = 11 µM and [OAA] = 1.9 µM. It is clear that ATP is the strongest and AMP the weakest inhibitor. In Figure 4D the measured flux is plotted as a function of [ACCOA] at [OAA] = 34 µM with ATP, ADP, and AMP present as indicated in the figure.

Figure 5 illustrates the affects of [Mg^2+^] and pH on inhibition by ATP, ADP, and AMP, and on overall catalytic activity. In Figure 5A the ATP-inhibition curve is plotted at different levels of free Mg^2+^ ion concentration. Mg^2+^ is shown to diminish the inhibition effect, suggesting that the species Mg·ATP^2−^ does not bind as significantly as ATP^4−^ and supporting our assumption that the free unbound species of ATP, ADP, and AMP are the important actors in competitive inhibition of the enzyme. Figure 5B plots the overall catalytic activity as a function of pH at fixed substrate concentrations, as indicated in the figure legend.

The data of Figures 4A, 4B, 4C, 4D, and 5B, were used to provide estimates of the kinetic parameters *K_mA_*, *K_mB_*, and *K_eA_* and the inhibition parameters *K_iATP_*, *K_iADP_*, *K_iAMP_*, and *K_iH_* for the liver enzyme. These estimates are listed in Table 2. The solid lines plotted in Figures 4 and 5 represent model predictions corresponding to these data sets. The data of Figure 5A were not used to estimate model parameters; thus the model predictions in Figure 5A represent validation of the overall model based on the prediction of how relative activity increases with [Mg^2+^] in the presence of ATP. Note that Shepherd and Garland reported a maximum in the plot of Figure 5A (their Figure 14) near [ATP] = 0.5 mM for [Mg^2+^] = 2 mM that is not captured by our model. (The [Mg^2+^] = 2 mM data are plotted as open circles in Figure 5A.) However, this apparent maxima is based on a single experimental data point; Smith and Williamson [17] report that they were unable to reproduce this observation.

Based on these data from Shepherd and Garland, we are able to determine that the likely site of ATP, ADP, and AMP binding is enzyme state 2—the complex E·OAA^−2^ in the proposed scheme of Equation (19). Alternative models, with nonproductive binding at states 1, 3, or 4, are not able to explain the observed data nearly as well as the model with inhibition at state 2. However, our model with a single inhibition site did not reproduce the data of Figure 7 of [15], which indicate that AMP may also act as a competitive inhibitor against OAA, while ATP and ADP do not. It may be possible to explain the AMP data from Figure 7 of [15] with a model that includes binding of AMP at both states 1 and 2. However, doing this would require increasing the complexity of the model based on a handful of unreplicated data points from a single study. The current model, in not including binding of ATP, ADP, or AMP to free enzyme (state 1), explains the consensus of the available data.

### Analysis of data for the heart enzyme from cow

The study of Smith and Williamson [17] allows us to test the validity compulsory-order ternary-complex mechanism model for data from citrate synthase obtained from bovine heart. Figure 6 plots data obtained from Figures 1 and 2 of [17], which allow us to estimate five parameters of the model for this isoform. Specifically, Figure 6A plots relative activity as a function of [ATP] at two different concentrations of OAA, as specific in the figure, and at fixed [ACCOA] = 16 µM. Figure 6B plots flux measured as a function of [ACCOA] at with different concentrations of the inhibitor ATP present and at fixed [OAA] = 5 µM. Finally, Figure 6C plots measured flux as function of [ACCOA] with [OAA] fixed at 3.1 µM and the inhibitor [SCOA] added to different concentrations as indicated in the figure. Experiments for this study were conducted at pH = 7.4 and at 21°C. As for the previous studies, we assume and overall ionic strength of 0.17 M, and [K^+^] = 100 mM, and [Mg^2+^] = 0.

Taken together, the data of Figure 6 provide the means to estimate kinetic parameters *K_mA_*, *K_mB_*, and *K_eA_* and the inhibition parameters *K_iATP_* and *K_iSCOA_* for the isoform of this enzyme from bovine heart. The solid lines plotted in the Figure correspond to the parameter estimates listed in Table 2.

### Sensitivity analysis

To estimate the sensitivity of the model prediction to finite changes in parameter values, the sensitivity was computed as the relative change in mean squared error due to a 10% change in a given parameter value. For each parameter estimate a sensitivity coefficient is defined as follows,
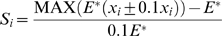
(25)where *E* ^*^ represents the minimum mean squared difference between model predictions and experimental data, and *x_i_* is the optimal value of the *i*th parameter. The term *E* ^*^(*x_i_*±0.1*x_i_*) is the error computed from setting parameter *x_i_* to 10% above and below its optimal value and reoptimizing all of the remaining parameter estimates. The sensitivity coefficients are listed in Table 2 in parenthesis following each parameter estimate. When the sensitivity coefficient is high, the data used to estimate that parameter value are able to provide a sensitive estimate of that parameter value. For the twenty parameter estimates reported in Table 2, three are associated with sensitivity coefficients of less than 1% and three others have sensitivity coefficients in the range 1–5%. Thus not all kinetic parameters are identified with high sensitivity.

### Alternative model

Previous analyses of the data used in this study have suggested a rapid-equilibrium random-order ternary-complex mechanism for citrate synthase [14], [15]. To provide an alternative to our proposed model and determine if the observed kinetics may be explained by this mechanism, we fit the data of Matsuoka and Srere [14] to the flux expression for the rapid-equilibrium random-order ternary-complex mechanism, given below [11]:
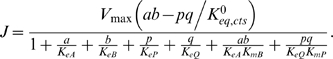
(26)Here we analyzed the Matsuoka and Srere data to avoid the complicating effects of inhibitors and because this data set is rich enough to exclude this model as a competing hypothesis.

**Figure 7:**
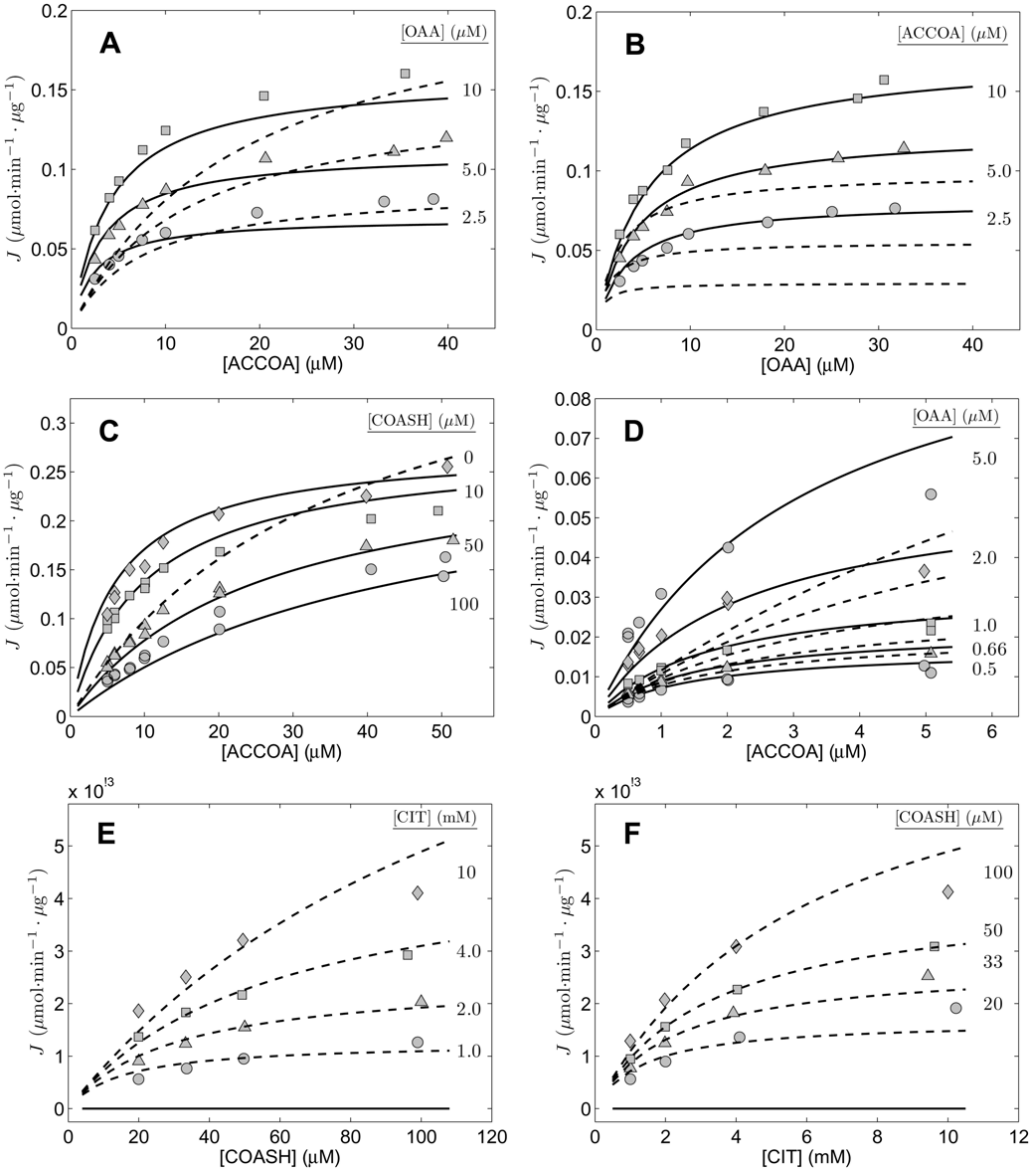
Analysis using random-order model of Equation (26). Data and conditions in A, B, C, and D are the same as for Figure 2. Data and conditions for E and F are the same as for Figures 3A and 3B, respectively Parameter values for solid line model predictions are *V_max_* = 0.320 µmol·min^−1^· µg^−1^, *K_mB_* = 6.20 µM, *K_mP_* = 8.00 µM, *K_eA_* = 1.35 µM, *K_eB_* = 1.10 µM, *K_eP_* = 21.6 nM, *K_eQ_* = 0.150 µM. Parameter values for dashed line model predictions are *V_max_* = 0.526 µmol·min^−1^·µg^−1^, *K_mB_* = 36.6 µM, *K_mP_* = 80.792 mM, *K_eA_* = 3.08 nM, *K_eB_* = 10.8 nM, *K_eP_* = 0.152 µM, *K_eQ_* = 17.0 µM.

Figure 7 replots the data of Figure 2 and 3 of the present study and illustrates two sets of fits using Equation (26), obtained by varying the seven adjustable parameters in this model. Parameter values are reported in the figure legend. The first fit (solid lines) is obtained as the best fit to the data in panels 7A through 7D on the forward operation of the enzyme. It is apparent that the forward flux data can be matched closely by the alternative model, at the expense of not being able to reproduce the reverse flux data of panel 7E and 7F. When the model is optimized to match both the forward and reverse data, the dashed-line model fits are obtained. In this case, none of the forward flux data are effectively matched. In particular, for the concentration range in Figure 7C, the dashed-line model fit shows no product inhibition by COASH, while the other panels at least reproduce the qualitative trends in the data. Since there is no parameter set that can reasonable match all of these data, this alternative model can be ruled out as inadequate.

## Discussion

Here we have introduced a model for the compulsory order ternary-complex catalytic mechanism that is formulated in terms of chemical species, allowing the model to account for variable state, including pH and metal ion concentrations. In addition, a general form of the model, with potential inhibitory binding at each enzyme state is introduced. The model is used to analyze independent data sets from a number of labs on different isoforms of citrate synthase and develop a consensus mechanism that explains the available data. This consensus mechanism provides a detailed understanding of the basic mechanism of this enzyme and can be useful in computational simulation of biochemical systems including this enzyme.

More generally, the basic model developed may serve as template for other two-substrate two-product reaction mechanisms. Given the depth and breath of the field of enzyme kinetics, it is surprising that the basic flux expression for the ordered ternary-complex mechanism with inhibition at all possible enzyme complexes has not been previously presented. Yet to our knowledge the flux expression introduced here has not been previously reported. Based on this general form, it is possible to systematically test models with inhibition at one or more potential sites and determine which model or models are consistent with the available data. In the case of citrate synthase examined here, the majority of data on inhibition by ATP, ADP, and AMP are best explained by nonproductive binding of these species to state 2 in the catalytic mechanism of Figure 1. Based on data at different pH values and with different concentrations of [Mg^2+^] in the media, we conclude that the unbound species of these reactants (ATP^4−^, ADP^3−^, AMP^2−^) are the species that bind to the enzyme complex.

The data analyzed here are not sensitive to distinguish the site of inhibition by SCOA. Models with binding to either enzyme state 1 or state 2 are equally able to explain the data. The parameters and model fits reported here correspond to the inhibition model of Equation (24) with binding to state 2.

Note that analysis of the data sets from isoforms from rat kidney, rat liver, and bovine heart does not provide a complete set of parameters for any of these isoforms. The data from the rat kidney isoform provide a detailed characterization of the basic kinetic parameters for this isoform, but without information on the inhibitors. The studies for the other isoforms did not probe the reverse reaction, therefore provide no information on kinetic parameters that appear only in the terms involving concentrations of products P and Q in the Equation (9). From cases where estimates of a given parameter are available for different isoforms (e.g., *K_mA_*, *K_mB_*, *K_eA_*) it is apparent that the estimates vary between the different species and/or tissue types from which the enzyme was obtained. Therefore, from these data a complete model parameterization for a specific species and tissue type is not possible.

It should also be noted that while we have ruled out the rapid-equilibrium random-order ternary-complex mechanism as an alternative model to explain observed data, our analysis does not rule out all possible alternatives. Rather, the model serves as a hypothesis that can explain, and is not disproved by, the observed data. While the data do not allow us to distinguish between certain alternative forms of the model, such as between the mechanisms of Equations (19) and (20), it is clear that widely reported mechanism for this enzyme is not able to explain the observed kinetics. Here the general equations for compulsory-order ternary-complex mechanism, with generalized inhibitor binding to any state in the catalytic cycle, are developed and shown to be consistent with the available data. This model will be useful in integrated modeling of biochemical systems involving this enzyme and in analyzing kinetic data from other enzymes thought to follow this general mechanism.
